# The enlargement of the maxillary ostium after balloon sinuplasty evaluated by a novel measuring technique from 3D CBCT images

**DOI:** 10.1038/s41598-021-03048-7

**Published:** 2021-12-08

**Authors:** Eerika Kalliomäki, Argyro J. Bizaki-Vallaskangas, Olli Valtonen, Markus Rautiainen

**Affiliations:** 1 Faculty of Medicine and Health Technology, Tampere University, Tampere, Finland; 2 Department of Otorhinolaryngology – Head and Neck Surgery, Tampere University Hospital, Tampere, Finland

**Keywords:** Anatomy, Infectious diseases, Three-dimensional imaging, Computed tomography, Quality of life

## Abstract

Our aim was to evaluate the effects of balloon sinuplasty on the size of the ostium in the maxillary sinuses in patients with chronic rhinosinusitis from cone beam computer tomography (CBCT) scans of the sinus. This is a blinded retrospective trial in patients who had undergone balloon sinuplasty of the maxillary sinus. CBCT scans were taken and SNOT-22 Quality of Life questionnaire completed before and 12 months after the operation. The size of the maxillary ostium was measured from the CBCT scans three-dimensionally. The association of changes in the SNOT-22 scores of the ostium was analysed. We discovered that the balloon sinuplasty increased the size of the maxillary ostium in all dimensions. The changes were statistically significant (*p*<0.05) in the axial diameter and the ostium area. The number of patent ostia increased after the intervention. The association between SNOT-22 score and ostium patency were statistically significant before the operation. Our conclusion is that the threedimensional measuring technique provides a reliable method to evaluate ostium dimensions. Balloon sinuplasty increased the size of the maxillary ostium and the result was maintained for 12 months after the operation.

## Introduction

Rhinosinusitis is a common problem worldwide that can have a remarkable effect on quality of life. Furthermore, it can develop into chronic rhinosinusitis which, in turn, can cause pathological changes to the mucosa of the nasal cavity. Chronic rhinosinusitis is primarily treated clinically, although some patients still need an operation to ease the symptoms^1,2^.

Endoscopic sinus surgery (ESS) is a standard treatment alternative for chronic rhinosinusitis that is effective and improves the quality of life of patients, whereas balloon sinuplasty is a less invasive technique that does not remove any tissue, but instead remodels it^3,4^. Balloon sinuplasty improves quality of life and is safe and effective in adults as well as in children^5–11^. Indeed, because balloon sinuplasty is a relatively atraumatic procedure, it is also a suitable treatment option for critically ill patients^12^. Some studies have indicated that balloon sinuplasty technique is as effective as ESS^6,13^.

Although considerable effort has been devoted to studying the effects of balloon sinuplasty using quality of life questionnaires, Lund-Mackay scoring, acoustic rhinometry and inflammation markers, rather less attention has been paid to the radiological changes of the maxillary ostium^4,6,8–10,12–16^. Consequently, it remains unclear whether the changes in the dimensions of the ostium are significant and constant. Instead of some endoscopic evaluations of ostium enlargement after balloon sinuplasty, the exact measurements of the maxillary ostium based on CT scans are missing^11^.

Of particular interest is the ostiomeatal complex, which is an intricate structure that varies between individuals and the different planes of computer tomography (CT) images^17^. We have found it difficult to find differences in the size of the ostium from CT images before and after balloon sinuplasty when evaluating CT images only with reviewing the scans but without precisely measuring the ostium. Therefore, measurements should be taken three-dimensionally, as using only one plane does not offer enough information.

In this study, the dimensions of the ostium have been directly measured three-dimensionally from radiological images taken before and after balloon sinuplasty. The aim was to investigate whether balloon sinuplasty intervention has any long-lasting effects on the dimensions of the maxillary ostium.

## Materials and methods

### Study design

This study is a blinded retrospective trial that was carried out in the Department of Otorhinolaryngology at Tampere University Hospital. Informed written consent and the approval of the Ethics Committee of the Pirkanmaa Hospital District were obtained prior to the first stage of the study. The research was performed in accordance with the relevant guidelines and regulations. The size of the maxillary ostium was measured from cone beam computed tomography (CBCT) scans of the paranasal sinuses. The images were measured before and after operation by one investigator who was unaware of the personal data and the timing of the imaging.

### Demographics

For this study, a total of 29 patients were analysed. All patients had chronic rhinosinusitis on the maxillary sinuses without major findings in other paranasal sinuses and had not previously undergone sinus operations (Table 1).

**Table 1:** Demographic information.

Number of patients	29
Mean age of patients ± SEM (yrs. Old)	38.17 ± 1.5
Sex of patients	11 males, 18 females
Smoking history (patients)	9
Usage of nasal steroids before surgery (patients)	22
Mean duration of symptoms (months) ± SEM	90 ± 12
Classification based on Lund-McKay score (unilateral score)	1–2: 21 pts
	3–4: 8 pts

SEM, Standard error of the mean.

The inclusion criteria were as follows: (a) patients had to have been diagnosed with chronic or recurrent rhinosinusitis of the maxillary sinus without severe pathology of other sinuses, (b) patients had to be older than 18 years and younger than 65 years, and (c) patients had to fulfill the indications for sinus surgery. Patients were excluded from the study if one of the following criteria was met: (a) previous sinus operations, (b) asthma, (c) acetylsalicylic acid (ASA)-intolerance, (d) diabetes or any other severe systemic disease, (e) visible polyps in nasal direct endoscopy, and (f) pregnancy at the time of enrolment to the study.

### Diagnosis

Routine diagnosis was based on patient history and direct endoscopic nasal examination. CBCT scans of the paranasal sinuses were acquired during the recruitment phase to evaluate the status of the paranasal sinuses. The diagnosis of rhinosinusitis was based on the criteria of EPOS^1^.

### Surgical procedure

For all patients, balloon sinuplasty was performed under local anaesthesia using 250 mg cocaine diluted in 5 ml of 0.1 mg/ml adrenaline. Also, 4 to 6 ml of 10 mg/ml lidocaine cum adrenaline solution was infiltrated to the mucosa of the uncinate process. Light sedation was achieved by the intravenous administration of 0.5 ml of 0.5 mg/ml alfentanil (Rapifen) and 0.5 ml of 1 mg/ml midazolam, but the patients remained conscious.

The cannulation and dilation of the sinus ostium was performed using a lighted guide wire called the Luma Sinus Illumination System. A flexible balloon (6 mm × 16 mm) was inflated up to 12 atm equal to 12 × 10^5^ Pa (Acclarent Inc, Menlo Park, CA, USA) for 1 min and the dilation was repeated once. Balloon sinuplasty was performed on both maxillary sinuses.

### SNOT-22 quality of life questionnaire

The quality of life and the symptoms of sinusitis were assessed before treatment as well as after treatment using the SNOT-22 questionnaire. The total SNOT-22 scores before treatment and at 12 months after treatment were used in this study.

### Measurement of the ostium

CBCT scans of the paranasal sinuses were taken prior to the operation and 12 months after. Each ostium and accessory ostium of the maxillary sinuses was measured blinded by using OnDemand3D software version 1.0 (CyberMed, Inc., Yuseong-gu, Daejeon, South Korea). The investigator did not know whether the CBCT scan was taken before or after the surgical intervention. In our study, only the maxillary ostium data were analysed. Two of our authors cooperated in the beginning of the measuring process to ensure the measurements were taken properly.

**Figure 1:**
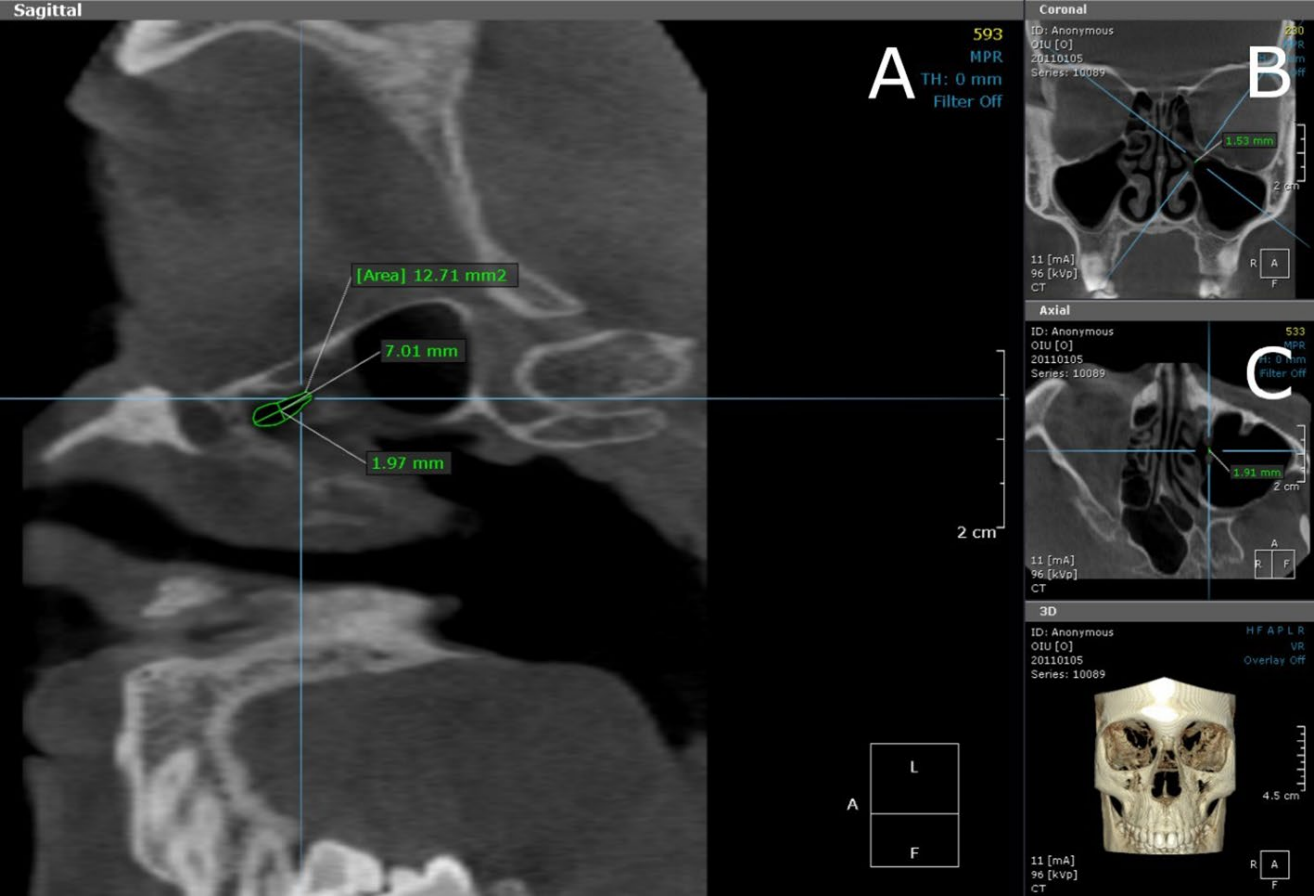
Measured diameters and area from sagittal (**A**), coronal (**B**) and axial (**C**) view. In the sagittal (**A**) and axial (**C**) view, the skull is turned three-dimensionally because the area and the diameter of the ostium is measured perpendicularly to the largest part of the coronal diameter in the coronal view (**B**). Blue lines indicate the direction and the fixing point of the ostium’s section and a white bordered cube indicates the position of the skull and the direction of the image. The letter symbols in the cube signify the following: H = Head view from superior angle, F = Foot view from inferior angle, A = Anterior view from anterior angle, P = Posterior view from posterior angle, L = Left view from left lateral angle and R = Right view from right lateral angle.

Ostium dimensions were examined and measured from the axial, coronal and sagittal views. The OnDemand3D allows CBCT scans to be examined three-dimensionally. This provides the most suitable visual field and is a reproducible technique for measuring the dimensions of the ostium. Viewing angles can be altered by rotating the skull. Each measuring plane of the ostium was determined individually in accordance with the perpendicular direction of the ostium (Fig. 1).

The first measurement of the CBCT image was taken from the widest part of the ostium which was estimated by viewing the coronal plane (Fig. 1). The coronal diameter was the fixing point for other measuring planes and made it possible to measure the axial diameter and ostium area perpendicularly from the widest part of the ostium. Measuring the area in the sagittal view was achieved by following the mucosal border and drawing freehand using the OnDemand3D software (Fig. 1). The area of the ostium in the sagittal view was drawn instead of calculating it from diameters, as not all ostia were circular. In some maxillary sinuses, the uncinate process and the wall of the maxillary sinus formed a canal-like structure, and therefore the size of the ostium was measured from the largest part of the lateral end of the canal.

Due to the measuring technique, which follows the mucosal surface, it was not possible to measure all of the ostia. In some cases, the mucosal oedema caused by inflammation in the maxillary sinuses concealed the precise geography of the ostium.

### Statistics

We used SPSS 26.0 software (IBM SPSS, Armonk, NY, USA) to analyse our data. The data did not comply with normal distribution according to Shapiro–Wilk test, and therefore we used the Wilcoxon signed-rank test to compare the dimensions of the ostium before and after the operation. SNOT-22 scoring in patients with obstructed and open ostia was analysed by Mann–Whitney U test.

## Results

### Measurement of the size of the maxillary ostium

Measurements were successfully taken from 84 patent ostia from the pre- and postoperative measurements of 29 patients. The number of patent ostia increased by 6 from the preoperative situation. From a total of 116 measured ostia, 32 were totally obstructed (Table 2). There were 22 ostia with enlargement and only 7 ostia with a shrinkage in all the three examined views in the 12 month follow up. 7 ostia were totally concealed due to the mucosal oedema before and 12 months after the operation and these were the only ostia without any change in the size of the ostium.

**Table 2:** The number of measured ostia (number of patients 29).

	Preoperative	Postoperative
Number of ostia	58	58
Number of totally obstructed ostia	19	13

The size of the ostium increased due to the surgical intervention in all dimensions. The changes in the maxillary ostium were statistically significant (*p* < 0.05) in the axial diameter and ostium area. A Wilcoxon signed rank test also indicated that the changes were statistically significant (Table 3).

**Table 3:** Median ostium dimensions of patent ostia before and after operation and the statistical significance of difference analysed with Wilcoxon signed ranks test between pre- and postoperative results.

	Before surgery (interquartile range)	After surgery (interquartile range)	Z	*p*
Coronal diameter (mm)	1.45 (2.27)	1.64 (1.39)	− 1.575^a^	0.12
Axial diameter (mm)	1.29 (2.16)	2.55 (2.99)	− 3.342^a^	0.001*
Sagittal view ostium area (mm^2^)	2.14 (6.31)	4.55 (11.43)	− 3.321^a^	0.001*

*These changes statistically significant (*p* < 0.05).

^a^Based on negative ranks.

### Patency of the ostium versus quality of life

**Figure 2:**
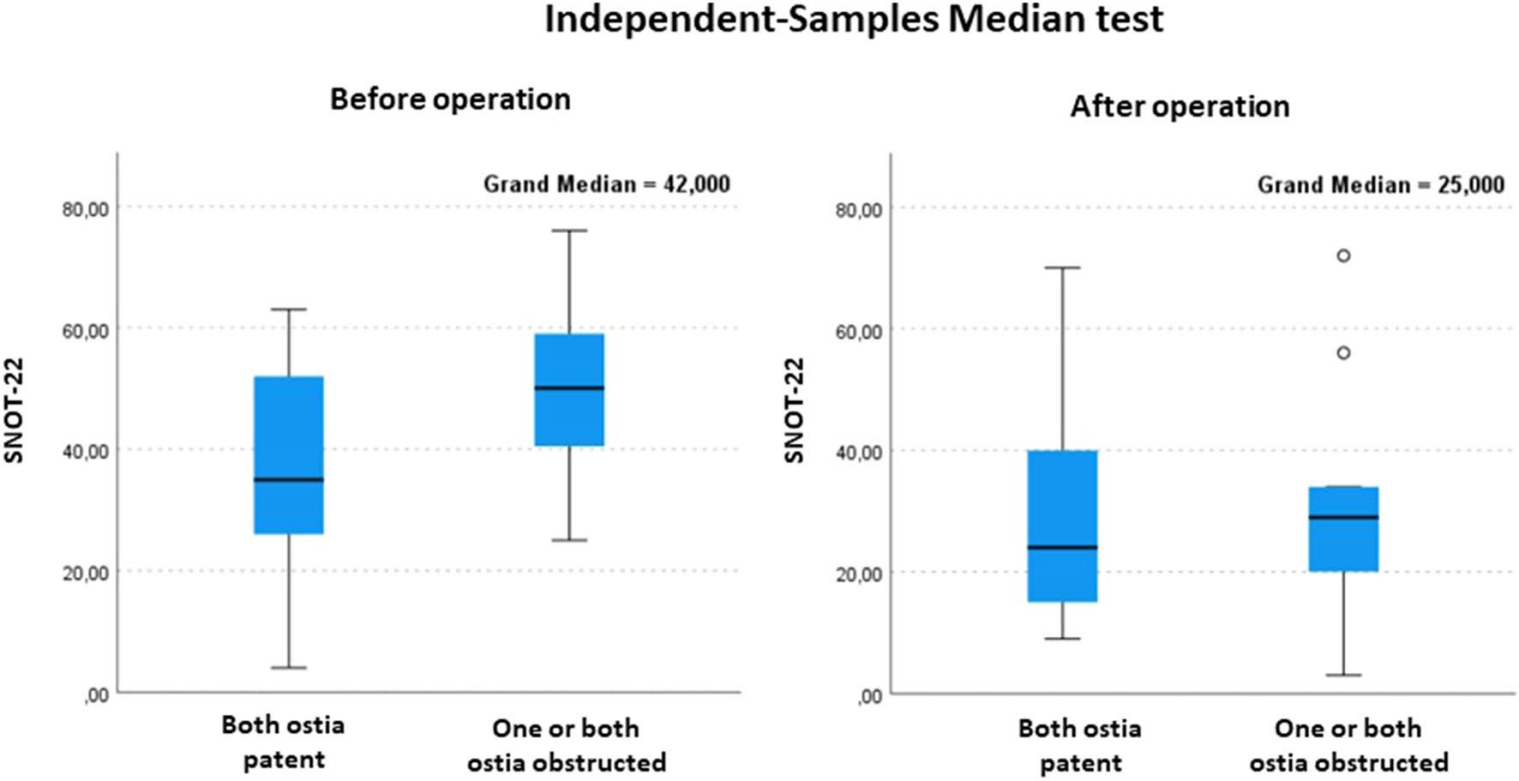
SNOT-22 scoring before and after balloon sinuplasty by Mann–Whitney U-test.

SNOT-22 score, analysed with Mann–Whitney U test, was higher when both or the other ostia were obstructed, and the difference was statistically significant (*p* < 0.05) before the operation. There was no statistically significant association after the operation (Fig. 2).

## Discussion

### Synopsis of key findings

Measuring the size of the maxillary ostium showed that the dimensions of the ostium increased after balloon sinuplasty intervention. Only the coronal diameter showed no statistically significant change.

In addition to the surgical effect of enlarging the size of the bony ostium, the reduction of inflammation in the mucosal membrane of the maxillary sinuses after the surgical intervention might also have had an influence on the size of the ostium^18^.

It must be acknowledged that the measurement of the dimensions of the ostium is challenging due to the complex structure of the ostium^17^. In addition, the intumescence of the mucosal membrane, the possible slightly different position of the head and the different plane of the CBCT scans complicates the measurements. To ensure that these challenges had only the slightest effect on the results, the measurements were taken from three different dimensions (coronal, axial and sagittal). The three-dimensional measurement increases the reliability of the ostium dimensions. Furthermore, our decision to measure the area of the ostium by drawing freehand strengthens the reliability because the morphology of the ostium varies among individual patients.

Additionally, SNOT-22 score was significantly higher in patients with one or both ostia obstructed than in patients with patent maxillary ostia before the operation. The size of patent ostia did not correlate with SNOT-22 score. This is probably because once the ostium is patent and allows drainage of the sinus, its size is no longer that crucial for its function^19^.

### Strengths of the study

This retrospective study was performed blinded to provide reliable information with a statistically sufficient sample size. The technique we have developed to examine the dimensions of the ostium from three different views provides a detailed and calculated observation of the ostium and produces more reliable results than two dimensional measurements which is in line with previous studies^20,21^. To minimize the bias of the measurement technique, each CBCT scan was measured by one investigator who was unaware of whether the CBCT scan was taken prior or after the balloon sinuplasty.

### Comparisons with other studies

We are unaware of any previous study that has examined the accurate dimension of the maxillary ostium by measuring it directly from CBCT scans. To date, most studies have only examined the effect of balloon sinuplasty intervention by using quality of life questionnaires, Lund-Mackay score, acoustic rhinometry, Lund-Mackay Computed Tomographic Scans of the nose and paranasal sinuses (CT-PNS) score, Diagnostic nasal endoscopy (DNE) score and inflammation markers^6,9–11,13,15,16,18,22–24^. Lund-Mackay scoring reduced in both studies that used CT-PNS and DNE scoring which could indicate similar results as in our study even though the inclusion and exclusion criteria were different and our study focused specifically on the size of the ostium than in the entire maxillary sinus^23,24^. Interestingly, one study measured the size and maintenance of the ostium after balloon sinuplasty, even though the aim of the study was to measure the drainage pathways of the frontal sinus^16^.

### Clinical applicability of the study

The results of this study clearly show a significant increase in ostium dimensions after balloon sinuplasty, and the effect was shown to last for at least 12 months. In addition, the amount of totally obstructed ostia decreased significantly. This information provides support for the more extensive use of the less invasive balloon sinuplasty technique.

The patients who took part in this study were without severe pathology of other sinuses. This ensured that changes in the maxillary ostium were the only significant finding. As a result, certain subgroups were excluded from the scope of this study, and the results are not therefore comparable to patients who have more severe findings in other paranasal sinuses.

Three-dimensional measuring technique improves spatial orientation and the accuracy of the measurements which ensures better repeatability. That could also indicate that it is not as necessary to repeat three-dimensional measuring as it is in two-dimensional measuring^20,21,25^.

## Conclusion

To date, the exact measurement of the size of the maxillary ostium has been challenging. However, in the present study, we have shown that by using our novel method, where viewing angles can be altered three-dimensionally, reliable measurements are possible.

This study has shown for the first time that balloon sinuplasty intervention increases the size of the maxillary ostium. Moreover, the enlargement was maintained for 12 months after the operation. Higher quality of life was associated with both ostia patent before balloon sinuplasty.
